# Supplementary material for: Hybrid Allosteric Modulators of M1 Muscarinic Receptors Enhance Acetylcholine Efficacy and Decrease Locomotor Activity and Turning Behaviors in Zebrafish
Source: Res Sq. 2024 Feb 15:rs.3.rs-3901189. Preprint. [Version 1] doi: 10.21203/rs.3.rs-3901189/v1 (PMC10896388; doi:10.21203/rs.3.rs-3901189/v1)
Supplement: Supplement 1 [file NIHPPRS3901189v1-supplement-1.pdf]

Supplementary Files

This is a list of supplementary files associated with this preprint. Click to download.

- Table2to4.docx
- SupplementalMaterials.pdf
